# MicroRNA-200c Attenuates the Tumor-Infiltrating Capacity of Macrophages

**DOI:** 10.3390/biology11030349

**Published:** 2022-02-22

**Authors:** Rebecca Raue, Ann-Christin Frank, Dominik C. Fuhrmann, Patricia de la Cruz-Ojeda, Silvia Rösser, Rebekka Bauer, Giulia Cardamone, Andreas Weigert, Shahzad Nawaz Syed, Tobias Schmid, Bernhard Brüne

**Affiliations:** 1 Institute of Biochemistry I, Faculty of Medicine, Goethe-University Frankfurt, 60590 Frankfurt, Germany; raue@biochem.uni-frankfurt.de (R.R.); frank@biochem.uni-frankfurt.de (A.-C.F.); fuhrmann@biochem.uni-frankfurt.de (D.C.F.); roesser@biochem.uni-frankfurt.de (S.R.); bauer@biochem.uni-frankfurt.de (R.B.); cardamone@biochem.uni-frankfurt.de (G.C.); weigert@biochem.uni-frankfurt.de (A.W.); syed@biochem.uni-frankfurt.de (S.N.S.); b.bruene@biochem.uni-frankfurt.de (B.B.); 2 Institute of Biomedicine of Seville (IBiS), Hospital University “Virgen del Rocío”/CSIC/University of Seville, 41013 Seville, Spain; patricia.cruz.ojeda@gmail.com; 3 German Cancer Consortium (DKTK), Partner Site Frankfurt, 60590 Frankfurt, Germany; 4 Frankfurt Cancer Institute, Goethe-University Frankfurt, 60596 Frankfurt, Germany; 5 Fraunhofer Institute for Translational Medicine and Pharmacology, 60596 Frankfurt, Germany

**Keywords:** macrophage, breast tumor, miR, tumor microenvironment

## Abstract

**Simple Summary:**

The tumor microenvironment determines the prognosis and outcome for cancer patients. Herein, tumor-associated macrophages are not only highly abundant, but also play a crucial role in shaping a tumor-supporting microenvironment. Both their recruitment to the tumor as well as their functional polarization toward a pro-tumorigenic phenotype are mediated by tumor-derived factors including microRNAs. However, the impact of most microRNAs on the tumor cell-macrophage crosstalk remains to be elucidated. Thus, we reached out to investigate the role of hsa-miR-200c-3p (miR-200c) in tumor cell–macrophage interactions, as it was shown to be differentially expressed during cancer progression and metastasis. miR-200c was highly expressed in MCF7 breast tumor cells compared to macrophages. Furthermore, we identified a CD36-dependent uptake of miR-200c, derived from apoptotic tumor cells, into macrophages. In macrophages, elevated miR-200c levels reduced the expression of numerous migration-associated mRNAs, consequently reducing the capacity of macrophages to infiltrate into tumor spheroids. Finally, a distinct signature of miR-200c-repressed, predicted targets was identified, which strongly correlated with tumor infiltration. Targeting the miR-200c transfer from dying tumor cells to macrophages might therefore provide the opportunity to specifically modulate tumor-associated macrophage recruitment.

**Abstract:**

Macrophages constitute a major part of the tumor-infiltrating immune cells. Within the tumor microenvironment, they acquire an alternatively activated, tumor-supporting phenotype. Factors released by tumor cells are crucial for the recruitment of tumor-associated macrophages. In the present project, we aimed to understand the role of hsa-miR-200c-3p (miR-200c) in the interplay between tumor cells and macrophages. To this end, we employed a coculture system of MCF7 breast tumor cells and primary human macrophages and observed the transfer of miR-200c from apoptotic tumor cells to macrophages, which required intact CD36 receptor in macrophages. We further comprehensively determined miR-200c targets in macrophages by mRNA-sequencing and identified numerous migration-associated mRNAs to be downregulated by miR-200c. Consequently, miR-200c attenuated macrophage infiltration into 3-dimensional tumor spheroids. miR-200c-mediated reduction in infiltration further correlated with a miR-200c migration signature comprised of the four miR-200c-repressed, predicted targets PPM1F, RAB11FIB2, RDX, and MSN.

## 1. Introduction

Despite diagnostic and therapeutic advances, breast cancer (BC) remains the most prevalent cancer worldwide and continues to account for a substantial number of tumor-associated deaths [1]. The progression of and prognosis for BC and other tumor entities is largely determined by the tumor microenvironment (TME) (i.e., by stromal cells including various immune cells and fibroblasts) [2,3,4]. Amongst the tumor-infiltrating immune cells, tumor-associated macrophages (TAM), predominantly derived from blood monocytes, play a crucial role. In fact, in BC, TAM may account for up to 50% of the tumor mass [5,6,7]. High numbers of infiltrating TAM further correlate with poor survival in most solid tumors [8,9]. While macrophages (MΦ) are initially a part of the anti-tumor response, their polarization changes to a pro-tumoral M2-like phenotype with anti-inflammatory and immunosuppressive properties within the TME [10,11]. Mechanisms contributing to the phenotype switch in MΦ were shown to involve environmental cues such as hypoxia and nutrient deprivation as well as factors released by apoptotic tumor cells including cytokines and lipid mediators [11,12]. Interestingly, tumor cells were also shown to affect surrounding stromal cells by releasing microRNAs (miRs) [13,14]. Along these lines, tumor-derived miRs are detectable in the serum of cancer patients and even serve as diagnostic biomarkers [15,16], since different tumor entities are characterized by specific miR expression profiles [17]. Tumors release miRs either packaged in extracellular vesicles such as microvesicles, exosomes, or apoptotic bodies [18] or complexed with proteins such as argonaute (Ago) proteins and high-/low-density lipoproteins (H/LDL) [19,20]. In addition to transferring miRs to MΦ, tumor cells also affect the synthesis or maturation of certain miRs in MΦ through receptor-mediated cascades, eventually modifying MΦ effector functions [21,22]. Of note, miRs are generally considered to (fine-)tune rather than establish entirely new cellular traits [23]. Thus, the effects of miRs often depend on already established or alternatively induced gene expression programs.

Previously, we observed elevated hsa-miR-200c-3p (from now on referred to as miR-200c) levels in MΦ after contact with apoptotic tumor cells [24]. miR-200c is a member of the miR-200 family, which encompasses miR-200a, miR-200b, miR-200c, miR-429, and miR-141 [25,26]. Various members of the miR-200 family were found in the serum of patients with metastatic BC and aberrant expression of miR-200c was associated with enhanced tumor progression, metastasis, and drug resistance in various tumor types including lung cancer, ovarian cancer, colorectal cancer, and BC [27,28,29,30,31]. miR-200c was further put forward both as a tumor prognostic biomarker [15,32] and as a potential therapeutic option for cancer patients [30,33]. With respect to the interplay between tumor cells and MΦ, there is evidence that triple negative breast cancer cells secrete elevated levels of plasminogen activator inhibitor 1 (PAI-1) and interleukin 10 (IL10) after the restoration of miR-200c levels, thereby supporting a tumor-promoting M2-like phenotype in TAM [34]. On the other hand, miR-200c overexpression in MΦ suppressed the tumor-associated switch from a pro-inflammatory to an anti-inflammatory, immune-modulatory TAM phenotype [35].

In light of these apparently contradictory findings on the role of miR-200c in MΦ, and considering elevated miR-200c levels in MΦ after coculture with tumor cells, we aimed to determine how miR-200c levels in MΦ increase upon contact with apoptotic tumor cells and what the functional consequences of elevated miR-200c in MΦ are.

## 2. Materials and Methods

### 2.1. Reagents

Cytochalasin D (CytD), carbenoxolone (CBX), sulfo-N-succinimidyl oleate sodium (SSO), and penicillin-streptomycin were ordered from Sigma-Aldrich (St. Louis, MO, USA). The monoclonal CD36 blocking antibody and corresponding IgG control were purchased from ImmunoTools (Friesoythe, Germany). CD36 short blocking peptide SLINKSKSSMF was synthesized as published previously [36]. All reagents were dissolved according to the manufacturer’s instructions.

### 2.2. Cell Culture

Human MΦ were maintained in RPMI 1640 medium containing 5% AB-positive human serum (DRK Blutspendedienst Baden-Württemberg-Hessen, Frankfurt, Germany), 100 U/mL penicillin, and 100 µg/mL streptomycin (MΦ media). Tumor cell lines were obtained from ATCC-LGC Standards GmbH (Wesel, Germany) and the media were supplemented with 10% fetal bovine serum (FBS; Capricorn Scientific GmbH, Ebsdorfergrund, Germany), 100 U/mL penicillin, and 100 µg/mL streptomycin. MCF7 cells were cultured in RPMI 1640 medium additionally containing 1% sodium pyruvate and 1% non-essential amino acids, while MDA-MB-231 cells were cultured in DMEM. All cells were cultured at 37 °C and 5% CO_2_ in a humidified atmosphere. Media were obtained from Thermo Fisher Scientific (Waltham, MA, USA) and all other cell culture supplements came from Sigma-Aldrich, if not indicated differently.

### 2.3. Generation of Apoptotic Cell-Conditioned Media from MCF7 Cells

Apoptotic cell-conditioned media (ACM) from MCF7 cells were generated by treating MCF7 cells at 90% confluency with 1 µg/mL staurosporine (STS; Sigma-Aldrich) for 1 h. After three washing steps with PBS, cells were cultured in serum-free RPMI 1640 medium for an additional 16 h. Final supernatants were collected, and cells were removed by centrifugation at 2000× *g* and 4 °C for 10 min. Subsequently, residual cellular contents and debris were eliminated by centrifugation at 4000× *g* and 4 °C for 10 min to allow for efficient microRNA isolation [37]. Viable cell-conditioned media (VCM) were generated accordingly without the apoptotic stimulus. For MΦ stimulation, VCM/ACM were diluted 1:1 in MΦ medium.

### 2.4. Generation of Human Macrophages from Buffy Coats

Buffy coats of anonymous healthy donors were obtained from DRK Blutspendedienst and peripheral blood mononuclear cells (PBMC) were isolated using Pancoll gradients (PAN Biotech, Aidenbach, Germany). After washing the cells twice with PBS, PBMC were seeded onto high-adherence culture dishes (Sarstedt, Nümbrecht, Germany) in RPMI 1640 medium supplemented with 100 U/mL penicillin and 100 µg/mL streptomycin. Non-adherent cells were washed away after 1 h and the remaining cells were maintained in MΦ medium for seven days to allow for differentiation to MΦ.

### 2.5. Macrophage Migration Assays

MΦ migration was assessed using a scratch assay and live cell tracking of MΦ on fibronectin. For the scratch assay, 1.5 × 10^5^ MΦ in 6-well plates were transfected with mimic control (MISSION^®^ miR negative control 2 (cel-miR-39a; Sigma-Aldrich)) or hsa-miR-200c-3p mimic (MISSION^®^ hsa-miR-200c-3p mimic). After 24 h, a 10 µL pipette tip was used to scratch the MΦ layer in a marked area. Pictures were taken every 24 h using a Canon EOS 600D camera (Canon, Ōta, Tokio, Japan) and a transmitted-light microscope (AxioVert 40; Zeiss, Oberkochen, Germany). The scratch area was evaluated using ImageJ software. To observe random MΦ migration on fibronectin, 2 × 10^4^ miR-200c mimic-transfected MΦ were transferred onto fibronectin (1.5 μg/cm^2^, fibronectin human plasma, Sigma Aldrich)-coated 8 well ibiTreat µ-slides (ibidi GmbH, Gräfelfing, Germany) 48 h after transfection. The coating was achieved following the manufacturer’s instructions. Live cell tracking was performed on a Cell Observer microscope (Zeiss) at 37 °C and 5% CO_2_ for 16 h with pictures taken every 10 min. Analysis of cell migration was performed using the tracking application of the AxioVisionSoftware with 50 cells tracked per condition of each replicate. Migration plots were generated for 15 representative tracks using the Chemotaxis and Migration Tool from ibidi.

### 2.6. Coculture of Primary Human Macrophages with MCF7 Cells or Spheroids

For coculture experiments, MΦ were cultured at a density of 1.5 × 10^5^ cells/mL. MCF7 cancer cells were added at the same density in MΦ media. Cocultures were maintained for the indicated times, before residual MCF7 cells were removed from the plates using trypsin–EDTA for 3–5 min, which left the adherence of MΦ unaltered.

MCF7 tumor spheroids were generated using the liquid-overlay technique as described previously [38]. In brief, 96-well plates were coated with 1% agarose (Sigma-Aldrich) and 5 × 10^3^ MCF7 cells were added per well. After centrifugation for 5 min at 500× *g* cells were allowed to form spheroids for four days. A sample of 5 × 10^4^ primary human MΦ was transferred to spheroids 48 h after transfection. After an additional 24 h, MΦ infiltration into MCF7 tumor spheroids was measured using flow cytometry.

### 2.7. RNA Isolation, Reverse Transcription, and Quantitative Real-Time PCR

RNA isolation from cells and cell culture supernatants was performed using TRIzol^®^ Reagent (Thermo Fisher Scientific) according to the manufacturer’s instruction with the exception that 30 µg GlycoBlue™ Blue Coprecipitant (Thermo Fisher Scientific) was added to the aqueous phase before RNA precipitation. Reverse transcription for mRNA expression analyses was performed using the Maxima First Strand cDNA Synthesis Kit for RT-qPCR (Thermo Fisher Scientific). For miR analysis, cDNA was transcribed using the MystiCq™ microRNA cDNA Synthesis Mix (Sigma-Aldrich) according to the manufacturer’s instructions. The QuantStudio 3/5 Real-Time PCR System and PowerUP™ SYBR™ Green Master Mix (both from Thermo Fisher Scientific) were used to perform real-time quantitative PCR (qPCR). Primers for SNORD44 came from Sigma-Aldrich and all other primers from Biomers (Ulm, Germany). Primer sequences are presented in Table 1. Relative mRNA/miR expression was calculated using the QuantStudio qPCR data analysis software (Thermo Fisher Scientific) and the ∆∆Ct method and was normalized to the indicated respective control RNAs.

### 2.8. miR Mimic and siRNA Transfection of Human Macrophages

miR mimic and siRNA were transfected using HiPerfect (Qiagen, Hilden, Germany) according to the manufacturer’s instructions. Overexpression of miR-200c in primary human MΦ was performed in 6-well plates using MISSION^®^ hsa-miR-200c-3p mimic or MISSION^®^ miR negative control 2 (cel-miR-39a; both from Sigma-Aldrich). To block NRP1/2 or CD36 gene expression, MΦ were transfected with ON-TARGETplus NRP1/2 siRNA, ON-TARGETplus CD36 siRNA, or control siRNA (all from Dharmacon, Lafayette, CO, USA).

### 2.9. Library Preparation, Quality Control, and mRNA Sequencing

For miR-200c target identification, RNA was isolated from miR-200c overexpressing MΦ 72 h after transfection using the RNeasy Plus Mini Kit (Qiagen). RNA quality was determined using the RNA ScreenTape on a TapeStation 4150 (Agilent Technologies, Santa Clara, CA, USA) and RNA concentration was measured with the Qubit™ RNA HS Assay Kit on a Qubit^®^ 3.0 Fluorometer (Thermo Fisher Scientific). Subsequently, library preparation was performed with the QuantSeq 3′ mRNA-Seq Library Prep Kit FWD with UDI for Illumina (Lexogen, Vienna, Austria). Library quality was assessed using High Sensitivity D1000 ScreenTape (Agilent Technologies) and after determination of the library concentration with the Qubit™ dsDNA HS Assay Kit, an equimolar lane mix of 2 nM was prepared. All kits were used according to the respective manufacturer’s instructions.

Single end sequencing was performed on the NextSeq2000 platform (Illumina, San Diego, CA, USA) with 100 cycles. Sequencing data were analyzed using the Bluebee QuantSeq Data Analysis Pipeline. Briefly, reads were quality and adapter trimmed using Bbduk from the bbmap suite before mapping against the human genome GRCh38 using STAR Aligner with modified ENCODE settings. HTSeq-count was used for gene read counting and differential expression analysis was performed using the DESeq2 pipeline. Subsequently, GO term analysis was carried out with GOrilla [39] using downregulated targets (log2-fold change ≤ −0.66). For target prediction, the miRDB [40,41] and TargetScanHuman 8.0 [42] databases were used. MiRDB predictions were filtered for a target score > 80 and TargetScan predictions for a cumulative weighted context++ score ≤ −0.4. Heatmaps were generated using Morpheus (https://software.broadinstitute.org/morpheus, accessed on 15 April 2021).

### 2.10. Flow Cytometric Analysis

For flow cytometry, 10 spheroids were pooled after MΦ infiltration and washed 3× with PBS to remove non-infiltrating MΦ. Single-cell suspensions were obtained by treatment of the spheroids with Accutase^®^ Solution (Sigma-Aldrich) for 40 min at 37 °C and subsequent dissociation by repeated pipetting. After centrifugation at 500× *g* and 4 °C for 5 min, 2% human FcR Blocking Reagent (Miltenyi Biotec, Bergisch Gladbach, Germany) in PBS with 0.5% BSA was applied for 15 min on ice to block nonspecific antibody binding to Fc-gamma receptors. Afterward, infiltrating MΦ were stained with Alexa Fluor^®^ 700 CD45 mouse anti-human antibody (1:100 dilution; BD Biosciences, Franklin Lakes, NJ, USA) for 20 min on ice in the dark. Analysis was performed on a FACSymphony™ A5 flow cytometer (BD Biosciences) using flow-count fluorospheres (Beckman Coulter, Brea, CA, USA) as an internal counting standard. Data were analyzed using the FlowJo software V10 (Treestar, Ashland, OR, USA).

### 2.11. Statistical Analysis

Statistical analyses were carried out using Graph Pad Prism 7. All data are presented as mean values ± SEM of at least three independent experiments. One-sample t-test was used for statistical analyses. Significant differences between experimental groups are indicated by asterisks (* *p* < 0.05, ** *p* < 0.01, *** *p* < 0.001). For correlation analysis, Pearson r values were calculated using Graph Pad Prism 7.

## 3. Results

### 3.1. miR-200c Increases in Human MΦ upon Coculture with Apoptotic MCF7 Cells

Since miR-200c is well established to play an important role in tumor progression, and our previous findings indicated that miR-200c increases in MΦ upon coculture with MCF7 breast tumor cells [24], we aimed to elucidate its role in the interplay between tumor cells and TAM.

**Figure 1:**
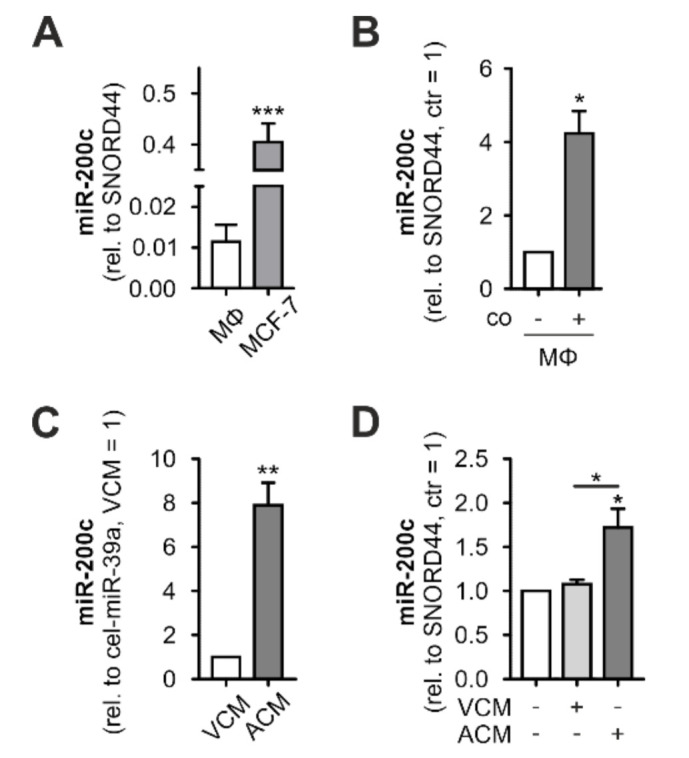
Interaction with apoptotic MCF-7 cells leads to increased miR-200c levels in primary human MΦ. miR-200c levels in (**A**) primary human macrophages (MΦ) and MCF7 cells, (**B**) MΦ after 48 h coculture (co) with MCF7 cells, (**C**) apoptotic or viable MCF7 cell-conditioned medium (ACM/VCM), and (**D**) MΦ 24 h after treatment with ACM/VCM (1 h). miR-200c levels were determined using qPCR and normalized to either SNORD44 (cellular levels) or spiked-in cel-miR-39a (CM). Data are depicted as mean ± SEM (*n* ≥ 4; * *p* < 0.05, ** *p* < 0.01, *** *p* < 0.001).

An initial analysis of miR-200c expression in MΦ and MCF7 cells revealed miR-200c expression in MΦ at very low levels, compared to MCF7 cells (Figure 1A). In line with previous observations, miR-200c significantly increased in MΦ after 48 h coculture with MCF7 cells (Figure 1B). In contrast, MDA-MB-231 breast tumor cells expressed lower basal miR-200c levels than MΦ and did not induce miR-200c expression in MΦ upon coculture (Figure S1). To assess if the increase after coculture with MCF7 cells might depend on MCF7 cells undergoing cell death in the coculture setting [43], we induced apoptosis in MCF7 cells using staurosporine (1 µg/mL for 1 h), collected the cell-free supernatants after 24 h, and analyzed miR-200c in this apoptotic cell-conditioned medium (ACM). In fact, while conditioned medium from viable cells (VCM) appeared to contain only traces of miR-200c, miR 200c levels were strongly elevated in ACM (Figure 1C). Moreover, MΦ cultured in ACM contained significantly higher miR-200c levels than MΦ cultured in VCM or regular medium (Figure 1D). Elevated miR-200c levels in MΦ upon culture in ACM suggested that no direct contact to MCF7 cells is required for this increase.

Taken together, these data indicate that miR-200c increases in MΦ only after contact with or exposure to supernatants of apoptotic tumor cells, which substantially express miR-200c.

### 3.2. miR-200c Is Transferred from Apoptotic MCF7 Cells to MΦ

**Figure 2:**
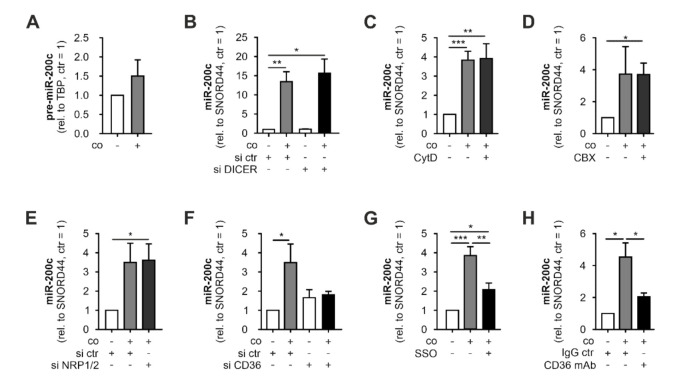
miR-200c is transferred from MCF7 cells to primary human MΦ. Primary human macrophages (MΦ) were cocultured with MCF7 cells before (pre-) miR-200c levels in MΦ were determined. (**A**) MΦ pre-miR-200c levels after coculture (48 h) with MCF7 cells. MΦ miR-200c levels upon (**B**) DICER knockdown, (**C**) cytochalasin D (CytD) treatment (10 µM), (**D**) carbenoxolone (CBX) treatment (100 µM), (**E**) neuropilin receptor (NRP) 1/2 knockdown, (**F**) CD36 receptor knockdown, (**G**) CD36 inhibitor sulfo-N-succinimidyl oleate (SSO) treatment (5 µM), or (**H**) neutralizing CD36 antibody treatment (2 µg/mL). (Pre-) miRNA levels were determined using qPCR and normalized to either SNORD44 or TBP (pre-miRNA). (**B**,**E**,**F**) Knockdown was achieved by transfection with specific siRNAs 24 h prior to coculture. Data are depicted as mean ± SEM (*n* ≥ 5; * *p* < 0.05, ** *p* < 0.01, *** *p* < 0.001).

We next aimed to determine the underlying mechanism of the miR-200c increase in MΦ upon contact with apoptotic tumor cells. To assess if enhanced de novo synthesis of miR-200c might account for elevated miR-200c levels in MΦ, we analyzed the expression of pre-miR-200c, which remained largely unaltered after coculture with MCF7 cells (Figure 2A). In line, efficient knockdown of the miR-processing enzyme DICER in MΦ (Figure S2; *left panel*) did not affect the coculture-induced miR-200c increase (Figure 2B). These observations ruled out enhanced miR-200c synthesis in the MΦ after contact with MCF7 cells, suggesting a transfer of miR-200c from apoptotic tumor cells to MΦ. Therefore, we aimed to decipher details of the miR-200c transfer. Initially, we inhibited cytoskeletal remodeling, which was shown to be required for the uptake of exosomes [44], by treating MΦ with cytochalasin D (CytD; 10 µM). However, CytD did not affect the miR-200c increase in MCF7-exposed MΦ (Figure 2C). As cell-to-cell transfer of miRs via gap junctions was previously proposed [45,46], we next used carbenoxolone (CBX; 100 µM) to block gap junctions. CBX treatment again did not alter miR-200c transfer from tumor cells to MΦ (Figure 2D), which corroborated earlier observations that cell-free ACM also enhanced miR-200c levels in MΦ (Figure 1D).

Since circulating miRs are commonly transported bound to proteins including argonaute proteins (AGO) [47,48] or low-/high-density lipoproteins (LDL/HDL) [49], we next tested the involvement of relevant receptors. To this end, we first knocked down NRP1/2 receptors, since these receptors have been proposed to facilitate the uptake of miR-AGO protein complexes [20]. However, the efficient knockdown of both NRP1 and 2 (Figure S2; *middle panels*) did not alter the increase in miR-200c in MΦ upon coculture (Figure 2E). An earlier analysis of circulating miRs in human plasma identified miR-200c to be present in LDL and HDL fractions [19]. Therefore, we next turned our attention to the scavenger receptor CD36, which, in addition to its established role in the uptake of fatty acids, phospholipids, and lipoproteins, was shown to be involved in the uptake of LDL-bound miRs [24]. Indeed, depletion of CD36 in MΦ using an siRNA approach (Figure S2; *right panel*) prevented the coculture-mediated increase in miR-200c in MΦ (Figure 2F). To further support this observation, we either pre-treated MΦ for 30 min with the irreversible CD36 inhibitor sulfo-N-succinimidyl oleate (SSO) or administered a monoclonal antibody against CD36 1 h before and during coculture with MCF7 cells. Similar to CD36 depletion by siRNA, inhibition of CD36 by SSO as well as by antibody blockage significantly reduced the coculture-dependent miR-200c increase in MΦ (Figure 2G,H). Our data indicate that miR-200c is transferred from apoptotic MCF7 cells to MΦ, and that the uptake into the latter requires the CD36 receptor.

### 3.3. Identification of a Migration-Associated miR-200c Target Gene Signature in MΦ

**Figure 3:**
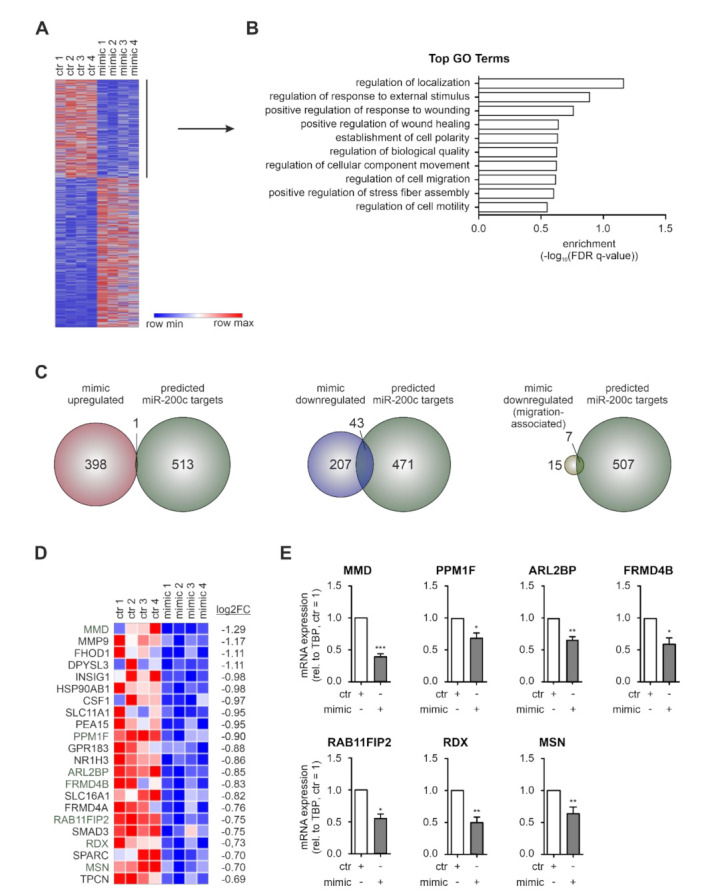
RNA-sequencing of miR-200c overexpressing MΦ identifies downregulation of migration-associated mRNAs. Primary human macrophages (MΦ) were transfected with miR-200c mimic (mimic) or mimic control (ctr) 72 h before mRNA was isolated for mRNA sequencing. (**A**) Heatmap of 649 differentially expressed genes in miR-200c-transfected MΦ (|log2FC| ≥ 0.66). (**B**) GO term analysis was performed with the 250 downregulated targets (log2FC ≤ −0.66) using GOrilla [39] and the top 10 processes (according to their *p*-value) are shown. (**C**) Venn diagrams intersecting predicted miR-200c targets (green) with targets upregulated (red; *left panel*) or downregulated (blue; *middle panel*) in mimic-transfected MΦ, and with targets downregulated in mimic-transfected MΦ associated with migration-related GO terms (yellow; *right panel*). (**D**) Heatmap depicting targets downregulated in mimic-transfected MΦ associated with migration-related GO terms (green: predicted miR-200c targets). (**E**) Expression of mimic downregulated, migration-associated, predicted miR-200c targets MMD (monocyte to macrophage differentiation-associated 1), PPM1F (protein phosphatase 1F), ARL2BP (ADP-ribosylation factor-like protein 2 binding protein), FRMD4B (FERM domain-containing 4B), RAB11FIP2 (RAB11 family-interacting protein 2), RDX (radixin), and MSN (moesin) was validated in MΦ 72 h after mimic transfection by qPCR. Data were normalized to ctr MΦ and are depicted as mean ± SEM (*n* ≥ 4; * *p* < 0.05, ** *p* < 0.01, *** *p* < 0.001).

Since miR-200c appears to be present in MΦ only after contact with apoptotic tumor cells, its effects on gene expression in MΦ have apparently been overlooked so far. Therefore, we overexpressed miR-200c in MΦ by transfection with miR-200c mimic (mimic) (Figure S3) and determined miR-200c-dependent gene expression changes by mRNA sequencing. Differential gene expression analysis identified 250 down- and 399 upregulated mRNAs (|fold change| ≥ 1.58) (Figure 3A, Table S1). To assess functional consequences of the presence of miR-200c in MΦ, GO term analyses were performed using GOrilla [39]. Considering that miRs repress rather than induce the expression of target mRNAs, only the downregulated candidates were included to identify potential direct effects. Interestingly, the analyses revealed a marked enrichment of cell mobility-related GO terms including regulation of localization, establishment of cell polarity, regulation of cell migration, positive regulation of stress fiber assembly, and regulation of cell motility (Figure 3B). In order to validate the direct impact of miR-200c on these functions, we compared gene expression changes upon miR-200c overexpression with a list of miR-200c targets predicted by miRDB (target score > 80) [40,41] and/or TargetScan (cumulative weighted context++ score ≤ −0.4) [42] (Table S2). Corroborating the notion that miRs downregulate direct targets, only one out of 399 upregulated targets (0.25%) emerged as a predicted miR-200c target, while 17.2% of the downregulated targets did so (Figure 3C). Of note, amongst the 22 confidentially downregulated targets (basemean > 50; p-adj ≤ 0.05) associated with migration-related processes (Figure 3D, Table S3), the proportion of predicted miR-200c targets was even higher (31.8%) (Figure 3C,D). Subsequently, miR-200c-dependent downregulation of the seven migration-associated, predicted miR-200c targets monocyte to macrophage differentiation-associated 1 (MMD), protein phosphatase 1F (PPM1F), ADP-ribosylation factor-like GTPase 2 binding protein (ARL2BP), FERM domain-containing 4B (FRMD4B), RAB11 family-interacting protein 2 (RAB11FIP2), radixin (RDX), and moesin (MSN) was successfully validated by qPCR analyses (Figure 3E).

Conclusively, miR-200c overexpression in MΦ promoted changes in gene expression with a marked enrichment within the downregulated targets of migration-associated processes, which contain a distinct signature of predicted miR-200c targets.

### 3.4. miR-200c-Induced Changes in Gene Expression Reduces Macrophage Infiltration

**Figure 4:**
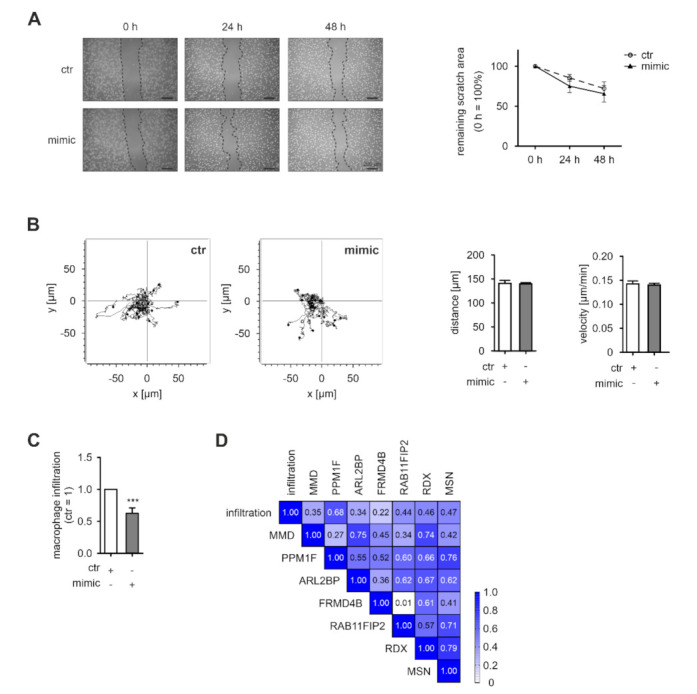
miR-200c overexpression in MΦ alters spheroid infiltration. Primary human macrophages (MΦ) were transfected with miR-200c mimic (mimic) or mimic control (ctr). (**A**) Twenty-four hours after transfection, a scratch was generated and scratch closure was evaluated after 24 and 48 h by microscopy (representative pictures shown in the *left panel*). Quantitative assessment of the remaining scratch area was carried out using ImageJ (*n* = 6; *right panel*). (**B**) Forty-eight hours after transfection, MΦ were seeded on fibronectin-coated ibiTreat µ-slides. MΦ migration was determined by live cell tracking for 16 h and quantified using the tracking application of the AxioVisionSoftware. Fifteen representative tracks are depicted (*left panel*) and a total of 50 cells per condition were analyzed per replicate regarding distance and velocity (*n* = 4, *right panel*). (**C**) Forty-eight hours after transfection, MΦ were transferred onto 4-day-old MCF7 tumor spheroids and allowed to infiltrate for 24 h prior to flow cytometric analysis. Infiltration of mimic-transfected MΦ into spheroids was normalized to ctr-transfected MΦ. Infiltration was determined as number of CD45+ cells in single cell suspensions of infiltrated tumor spheroids. Data are presented as mean ± SEM (*n* = 13; *** *p* < 0.001). (**D**) Correlation of tumor spheroid infiltration with the expression of potential miR-200c targets of the migration-associated miR-200c signature including MMD (monocyte to macrophage differentiation-associated 1), PPM1F (protein phosphatase 1F), ARL2BP (ADP-ribosylation factor-like protein 2 binding protein), FRMD4B (FERM domain-containing 4B), RAB11FIP2 (RAB11 family-interacting protein 2), RDX (radixin), and MSN (moesin). mRNA expression in MΦ was determined at the beginning of the infiltration.

Having established that miR-200c overexpression reduces the expression of a set of pro-migratory, potential miR-200c targets, we assessed whether these changes suffice to reduce MΦ migration. miR-200c mimic-transfected MΦ closed a freshly generated scratch at similar rates as the control MΦ (Figure 4A). Moreover, miR-200c overexpressing MΦ showed no changes in migration distance or velocity compared to the control-transfected MΦ on fibronectin-coated slides as determined by live cell tracking (Figure 4B).

Taking into account that miRNAs are considered as modulators rather than prime regulators of gene expression, the functional consequences of altered miRNA levels can be predicted to rather alter responses also induced by other factors. Therefore, we next evaluated the impact of miR-200c overexpression in a more complex migration context (i.e., we followed infiltration of miR-200c overexpressing MΦ into tumor spheroids of MCF7 cells). Indeed, we found that the spheroid infiltrating capacity of miR-200c mimic-expressing MΦ was significantly reduced compared to the control MΦ (Figure 4C and Figure S4). To further substantiate the contribution of the proposed migration-associated miR-200c target signature for the tumor infiltration phenotype of MΦ, we determined the correlation between the expression levels of the respective signature targets and MΦ infiltration. The correlations between the expression levels of PPM1F, RAB11FIB2, RDX, and MSN suggested a co-regulation of these factors (Figure 4D). Moreover, these four signature candidates also correlated with infiltration into spheroids (*r* = 0.44–0.68). In contrast, MMD, ARL2BP, and FRMD4B only correlated partially with other signature members, and also showed little correlation with infiltration (*r* = 0.22–0.35), even though MMD showed the strongest downregulation within the migration-associated cluster (Figure 3D,E).

In summary, our data suggest that miR-200c expression in MΦ reduces the expression of a broad set of migration-associated, predicted targets. A distinct subset of these genes appears to be tightly co-regulated and correlates with the capacity of MΦ to infiltrate into tumor spheroids.

## 4. Discussion

**Figure 5:**
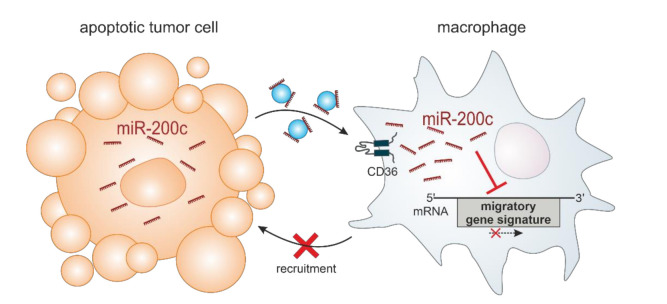
Model of the role of miR-200c in the interaction between tumor cells and MΦ. miR-200c is released from apoptotic, miR-200c-expressing tumor cells and taken up by MΦ via the CD36 receptor. In MΦ, miR-200c reduces the expression of a migration-associated gene signature, consequently limiting the tumor-infiltrating capacity of the MΦ.

miRs are important modulators of gene expression, affecting the cellular expression of their respective targets by RNA degradation and translational repression [50]. While the impact of many miRs has been characterized in various cell types, there is increasing evidence that miRs can also be released from their producing cells, provoking changes in recipient cells [13,28,51]. In this study, we characterized the transfer of miR-200c from apoptotic MCF7 breast tumor cells to MΦ and identified CD36 as a critical receptor for its uptake. In MΦ, miR-200c induced pronounced mRNA expression changes, specifically reducing the expression of migration-associated genes. Within this gene cluster, a signature of four potential miR-200c target genes emerged, which correlated with the capacity of MΦ to infiltrate into tumor spheroids (Figure 5).

miR-200c and other members of the miR-200 family were previously described to suppress epithelial to mesenchymal transition (EMT) of tumor cells by targeting the transcriptional repressors ZEB1 and ZEB2 [52,53]. In line, reduced miR-200c expression in breast tumors predicts poor patient survival [54]. In contrast to the primary tumor, miR-200c is upregulated at the metastatic site where it contributes to the reverse MET [55]. Altered miR-200c expression in the course of tumor progression is further underscored by the observation that triple negative breast tumors express lower miR-200c levels compared to less advanced, ER positive tumors [56], which is also reflected by the markedly lower miR-200c levels in advanced MDA-MB-231 cells compared to less invasive MCF7 cells [57].

However, the impact of miR-200c in the tumor context is not limited to the tumor cells. Reduced miR-200c expression in fibroblasts upon the interaction with tumor cells resulted in a switch toward a pro-tumoral fibroblast phenotype [29,58]. In contrast, in myeloid-derived suppressor cells (MDSC), tumor-derived signals (e.g., GM-CSF) induced miR-200c expression, which, by targeting PTEN and FOG2, enhanced the immuno-suppressive function of the MDSC, thereby exerting pro-tumorigenic functions [59]. Similarly, we observed an increase in miR-200c in MΦ after coculture with MCF7 breast tumor cells. We ruled out enhanced de novo synthesis of miR-200c in MΦ and identified the transfer of miR-200c from apoptotic tumor cells to MΦ as the underlying principle. This increase occurred only upon interaction with miR-200c-expressing MCF7 breast cancer cells, but not with more advanced MDA-MB-231 cells that contain negligible amounts of miR-200c. It can be speculated that the miR-200c transfers to MΦ and the resulting effects in TAM might be of relevance only during early, less invasive breast tumor stages. Moreover, as the release of miR-200c was only observed from apoptotic tumor cells expressing sufficient miR-200c levels already under basal conditions, it can be speculated that while miR-200c expression levels in tumor cells determine the releasable amount of miR-200c, apoptosis represents the releasing stimulus. Thus, in addition to tumor cell apoptosis due to insufficient nutrient or oxygen supply, or upon contact with immune cells, chemotherapy-induced cell death can be predicted to further enhance miR-200c-dependent repression of MΦ recruitment, if the affected tumor cells express relevant miR-200c levels. Of note, high miR-200c serum levels were previously shown to correlate with poor response to chemotherapy, however, the functional consequences of elevated serum miR-200c remained elusive [60].

Exchange of miRs between different cells is a widely observed phenomenon, which appears to be of particular importance in the tumor context, where miRs are transferred from tumor cells to stromal cells and vice versa [61,62,63]. The relevance of tumor-derived miRs in the TME and beyond is further underlined by the increasing number of miRs detected in the serum of tumor patients, serving both as biomarkers but also as potential therapeutic targets [16,64]. miRs are transported in the extracellular space either in extracellular vesicles including microvesicles, apoptotic bodies, and most prominently exosomes, or bound to proteins [13]. Along these lines, miR-200c was shown to be transported in extracellular vesicles from metastatic cells to non-metastatic BC cells, conferring metastatic traits to the latter [28]. In contrast, in the close interaction of MΦ with apoptotic tumor cells, we observed a CD36-dependent uptake of miR-200c into MΦ, as recently also shown for miR-375 [24]. Taking into account that CD36 serves as a receptor for the uptake of LDL, our observations nicely corroborate a previous report indicating that miR-200c in patient serum is not only found in the exosome fraction, but also bound to LDL and HDL [19].

While numerous studies have addressed the impact of MΦ-intrinsic miRs on MΦ function and phenotype [65,66,67,68], the functional consequences of miRs that are not or only minimally expressed in MΦ have remained largely elusive. In line with the notion that single miRs affect numerous targets by reducing their RNA stability via recruitment of the decay machinery to bound targets [69,70], 250 mRNAs appeared to be downregulated by miR-200c in MΦ. Interestingly, an even higher number of mRNAs increased, which corroborates previous reports that miR-200c activates important signaling cascades (e.g., PI3K) by targeting repressors of the respective cascades (e.g., PTEN) [59]. Similar to the function in tumor cells, miR-200c downregulated several mRNAs associated with migration-related processes. While miR-200c overexpression did not affect migration of MΦ in 2D assays, it markedly reduced the infiltration of MΦ into 3D tumor spheroids. In line with this, miR-200c expression in T11 tumors in vivo was shown to reduce the number of tumor-infiltrating macrophages [71]. Furthermore, our findings are in agreement with the concept that miRs fulfill modulatory functions rather than acting as solitary regulators of certain traits [23].

Amongst the migration-associated mRNAs downregulated by miR-200c overexpression, a subgroup was also predicted as putative miR-200c targets in silico. Within this group, a signature of four potential target genes (PPM1F, RAB11FIP2, RDX, MSN) was identified. Their expression correlated with the capacity of MΦ to infiltrate breast tumor spheroids in the context of the miR-200c-dependent reduction in MΦ infiltration. Previously, miR-375 was shown to be transferred to MΦ in the same context, but in contrast to miR-200c, miR-375 enhanced spheroid infiltration via repression of the migration-inhibitory factors tensin 3 and paxillin [24]. These apparently contradictory findings underscore the multitude of counteracting mechanisms allowing for the (fine-)tuning of tumor infiltration already in a simplified tumor spheroid model. Taking into account the complexity of regulatory processes and considering the modulatory function of miRs, it is neither surprising that miR-200c overexpression reduced spheroid infiltration by only 35% nor that the miR-200c-regulated migration signature only moderately correlated with the infiltration phenotype. Amongst the genes of the proposed miR-200c MΦ migration signature, PPM1F and MSN were already established as miR-200c targets, altering the motility of breast tumor cells [72,73]. Thus, an involvement in miR-200c-mediated MΦ migration seems plausible. RAB11FIP2 and RDX, on the other hand, emerged as novel miR-200c target candidates. RDX is functionally closely linked with MSN as part of the ezrin-RDX-MSN axis, which plays a role in cell adhesion, polarity, and thus eventually, cell migration [74,75]. Similarly, RAB11FIP2 regulates actin dynamics and has been shown to contribute to metastasis in colorectal cancer [76,77,78].

Since high TAM counts correlate with tumor progression and poor survival [8,9], our finding that miR-200c attenuates MΦ recruitment into tumors suggests an anti-tumorigenic function of miR-200c in the TME. Similarly, ectopic miR-200c expression was previously shown to support an anti-tumoral T-cell phenotype in adoptive T-cell therapy models [79]. In line with this, miR-200c-containing nanoparticles were recently shown to suppress tumor progression by altering the TME in a triple negative breast cancer model with low basal miR-200c expression [33]. In sharp contrast, elevated tumor miR-200c was shown to enhance the recruitment of immunosuppressive neutrophils, conferring resistance to immune checkpoint inhibitors in a subtype of triple negative breast cancers [71]. These findings highlight that it is critical to take miR-200c expression both in releasing tumor as well as in potential recipient stromal cells into account when considering potential miR-200c modulating interventions.

## 5. Conclusions

In the present study, we provide evidence for the transfer of miR-200c from apoptotic tumor cells to MΦ. In MΦ, miR-200c confers migration-regulatory properties by repressing expression of a specific signature of migration-associated targets. As miR-200c expression in tumor cells changes during tumor progression, the transfer of miR-200c from tumor cells to MΦ might add to synchronize a co-migratory behavior of tumor cells and MΦ. Further studies are needed to reveal the potential of miR-200c as a therapeutic target to interfere with the presence and migration of MΦ in the tumor context.

## Figures and Tables

**Table 1:** Human primers used for qPCR analysis.

Gene Name	Forward (5′–3′)	Reverse (5′–3′)
TBP	GGGCCGCCGGCTGTTTAACT	AGCCCTGAGCGTAAGGTGGCA
DICER	TGCTATGTCGCCTTGAATGTT	AATTTCTCGATAGGGGTGGTCTA
CD36	AAGCCAGGTATTGCAGTTCTTT	GCATTTGCTGATGTCTAGCACA
NRP1	GGCGCTTTTCGCAACGATAAA	TCGCATTTTTCACTTGGGTGAT
NRP2	GCTGGCTATATCACCTCTCCC	TCTCGATTTCAAAGTGAGGGTTG
hsa-miR-200c-3p	TAATACTGCCGGGTAATGATGGA	
pre-hsa-miR-200c-3p	TCGTCTTACCCAGCAGTGTTT	CTCCATCATTACCCGGCAGTAT
MMD	ATGCGTTGGTTTATCTGGCTC	AGTCCATCGGTGTTGTTCATTG
PPM1F	GGGATTCCCAGGTCATTTTGG	TCCTGCCGTTCTGGTCTGT
ARL2BP	GACGCCTTAGAAGGAGAGAG	TCCAACCACAGCATCAAATTC
FRMD4B	GATCACCGAGTTCTTGACCAC	TGGTTTCGCTCTCTACTTCGAT
RAB11FIP2	GCCTCTTTCGAGCTACCTGG	GATTGCCACCTGCCCTAAAAA
RDX	AATCGACAAAAAGGCACCTGA	CCATACATAAGGCCAAAATCCGC
MSN	ATGCCCAAAACGATCAGTGTG	ACTTGGCACGGAACTTAAAGAG

## Data Availability

The sequencing data presented in this study are openly available in GEO under accession number GSE195587.

## Supplementary Materials

The following supporting information can be downloaded at: https://www.mdpi.com/article/10.3390/biology11030349/s1, Figure S1: miR-200c expression in MDA-MB-231 cells; Figure S2: Knockdown efficiencies in macrophages; Figure S3: miR-200c overexpression in macrophages; Figure S4: FACS gating strategy; Table S1: Differentially expressed genes in miR-200c mimic-transfected primary human macrophages; Table S2: Predicted miR-200c targets; Table S3: Migration-associated targets reduced in miR-200c mimic-transfected primary human macrophages.
