# Supplementary material for: MicroRNA-200c Attenuates the Tumor-Infiltrating Capacity of Macrophages
Source: Biology (Basel). 2022 Feb 22;11(3):349. doi: 10.3390/biology11030349 (PMC8945044; doi:10.3390/biology11030349)
Supplement: Supplementary file 1 [file biology-11-00349-s001.zip › Raue et al - Supplementary Figures.pdf]

microRNA-200c attenuates the tumor-infiltrating capacity of macrophages

Rebecca Raue, Ann-Christin Frank, Dominik C. Fuhrmann, Patricia de la Cruz-Ojeda, Silvia Rösser, Rebekka Bauer, Giulia Cardamone, Andreas Weigert, Shahzad Nawaz Syed, Tobias Schmid, Bernhard Brüne

SUPPLEMENTARY FIGURES

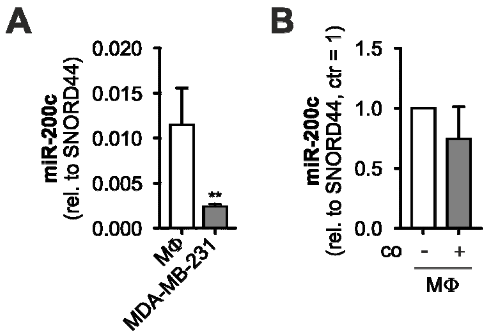

Figure S1. miR-200c expression in MDA-MB-231 cells. MiR-200c levels in (**A**) primary human macrophages (MΦ) and MDA-MB-231 cells, and (**B**) MΦ after 48 h coculture (co) with MDA-MB-231 cells. miR-200c levels were determined using qPCR and normalized to SNORD44. Data are depicted as mean ± SEM ($n \geq 3$; $^{**}p < 0.01$).

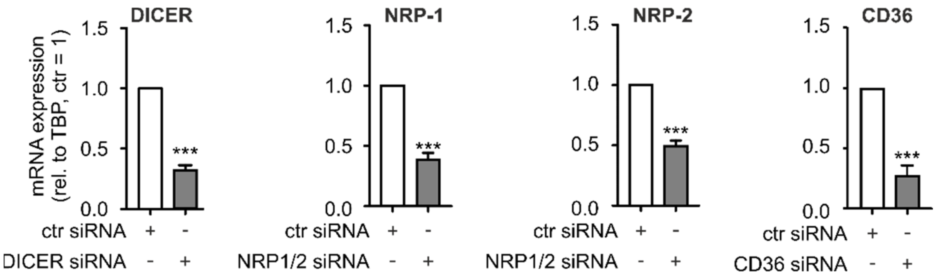

Figure S2. Knockdown efficiencies in macrophages. Primary human macrophages (MΦ) were transfected with specific siRNA to knockdown DICER, neuropilin (NRP) 1/2, or CD36. Knockdown efficiency was determined via qPCR 72 h after transfection. Data are normalized to control (ctr) si-transfected MΦ and are depicted as mean ± SEM ($n \geq 4$; ***$p < 0.001$).

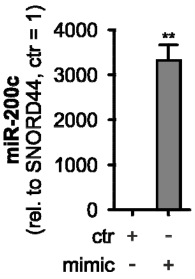

Figure S3. miR-200c overexpression in macrophages. Primary human macrophages (MΦ) were transfected with miR-200c mimic (mimic) or mimic control (ctr). 72 h after transfection the expression of miR-200c was analyzed via qPCR. Data are normalized to mimic control-transfected MΦ and are depicted as mean ± SEM (n = 4; **p < 0.01).

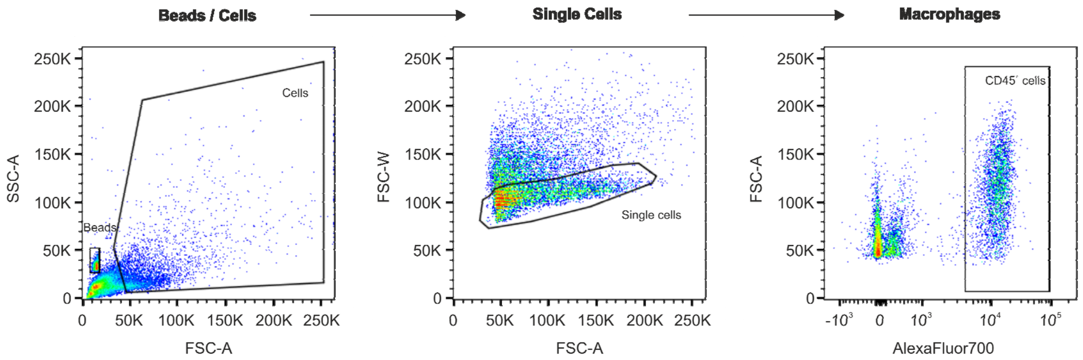

Figure S4. FACS gating strategy. For the quantification of the infiltration of primary human macrophages (MΦ) into MCF7 tumor spheroids, tumor spheroids infiltrated for 24 h with MΦ, were dissociated, single cell suspension cells were stained with an AlexaFluor700-coupled CD45 antibody, and single, CD45+ cells were counted relative to an internal counting standard (beads).
